# Translation, cross-cultural adaptation and psychometric validation of the self-efficacy scale for physical education teacher education majors toward children with disabilities for Brazilian Portuguese

**DOI:** 10.3389/fpsyg.2026.1782205

**Published:** 2026-04-20

**Authors:** Renato de Carvalho Guerreiro, Ciro Winckler, Raphael Moreira de Almeida, Martin E. Block, Andressa Silva

**Affiliations:** 1 Universidade Federal de Juiz de Fora – UFJF, Governador Valadares, Brazil; 2 Universidade Federal de Minas Gerais – UFMG, Belo Horizonte, Brazil; 3 Comitê Paralímpico Brasileiro, São Paulo, Brazil; 4 Universidade Federal de São Paulo – UNIFESP, São Paulo, Brazil; 5 University of Virginia, Charlottesville, VA, United States

**Keywords:** cross-cultural adaptation, EAE-EFI/Brazil, inclusive physical education, physical education teacher, professional development, SE-PETE-D, self-efficacy scale, special educational needs

## Abstract

**Introduction:**

The participation of people with disabilities (PWD) in physical activity and sports is essential for social inclusion and health promotion. The self-efficacy perceived by professionals greatly influences the success of inclusive practices; higher confidence in adapting activities and addressing diverse needs leads to better experiences for PWD. The self-efficacy scale for physical education teacher education majors toward children with disabilities (SE-PETE-D) measures this construct but lacked a validated Brazilian version.

**Objective:**

To translate, culturally adapt, and validate the Brazilian Portuguese version, named EAE-EFI/Brazil.

**Results:**

Content validity indices exceeded 0.90 across all subscales. Exploratory factor analysis revealed high internal consistency (Cronbach’s *α* > 0.96). Confirmatory factor analysis showed acceptable model fit (RMSEA <0.10; CFI, TLI, IFI > 0.90), supporting the original structure for subscales related to physical disability (PD) and visual impairment (VI).

**Conclusion:**

The EAE-EFI/Brazil is a valid and reliable tool for assessing physical education teachers’ and students’ self-efficacy concerning inclusion of individuals with intellectual disabilities, PD, and VI in Brazil. Future studies should evaluate the scale’s applicability in diverse contexts and explore links with professional development and institutional support, advancing inclusive practices and policies in Brazilian education.

## Introduction

The demographic landscape of Brazil reveals the significant size of the population of people with disabilities (PWD). The 2022 Demographic Census indicates that 8.9% of the population (18.6 million Brazilians) reported having some form of impairment (IBGE, 2023). This substantial population group necessitates effective public policies that guarantee their rights and promote their full inclusion in society. Within the education system, the inclusion of PWD has been a constitutionally guaranteed principle since 1988 (Art. 205, 208, 227), ensuring the right to inclusive education at all levels and modalities, with adaptations, accessibility, and specialized support (Law No. 13146/2015). These actions observed in Brazil are similar to those offered as part of the inclusive policy in the United States of America, which, through the Individuals with Disabilities Education Act (IDEA) of 2004, stipulates that students with disabilities should be educated alongside their non-disabled peers to the greatest extent possible (Wilson et al., 2020). These and other international experiences offer valuable insights for enhancing inclusive practices.

Despite regulatory advancements and growing awareness of inclusion, PWD in Brazil still face significant challenges. Recent data indicate higher rates of illiteracy and lower levels of employment and workforce participation in this population (PNAD, 2023). Furthermore, children with disabilities are at an increased risk of comorbidities due to limited engagement in everyday activities (Townsend, 1997). In this context, participation in sports and physical activities emerges as a crucial determinant of health and development for PWD (Martin Ginis et al., 2021). Inclusive physical education can offer benefits, such as improved levels of physical activity outside of school (Capio et al., 2015), motor development, wellbeing, self-esteem, and autonomy (da Rosa Romero and Carmona, 2017), as well as optimization of metabolic, physical, cognitive, mental, and social aspects, thereby enhancing the overall quality of life of PWD (Rimmer and Marques, 2012).

Despite the increasing emphasis on inclusive education and the creation of environments that foster the participation of all students, respecting their individual needs and abilities (Wilson et al., 2020), significant barriers persist in the inclusion of PWD in sports activities, both within and outside the school setting. These barriers include inadequate teacher training and professional development, limited support, overcrowded classrooms, excessive administrative demands, time constraints, and educators’ perceived lack of competence in teaching students with disabilities (Konza, 2008). Guaranteeing access for PWD to adapted physical activity hinges on the availability of qualified professionals (An and Meaney, 2015) and the existence of supportive environments, equipped with the resources necessary for effective inclusion (DeMatthews et al., 2025).

In this context, teacher self-confidence is a key factor for the successful inclusion of PWD in both physical education classes and, more broadly, in sports activities (Block and Rizzo, 1995). Perceived self-efficacy represents a specific form of self-confidence related to specific tasks and situations, understood as the belief in one’s ability to act and achieve a determined outcome effectively (Bandura, 1997). Self-confidence can influence, for example, how a teacher addresses the challenge of including a student with a disability in a physical education class. Teachers with higher levels of self-efficacy may be more inclined to implement adaptations to include a student with a disability (Nowland and Haegele, 2023). In addition, it has been demonstrated that appropriate training for working with PWD improved teachers’ perceptions of self-efficacy, indicating that training is an important element for effective and efficient professional performance (Taliaferro et al., 2015).

Considering the aforementioned, the “Self-Efficacy Scale for Physical Education Teacher Education Majors toward Children with Disabilities” (SE-PETE-D) was developed (Block et al., 2013) to assess the perceived self-efficacy of professionals in including individuals with intellectual disabilities (ID), physical disabilities (PD), and visual impairments (VI). The SE-PETE-D represented an advancement over the initial work of Hutzler et al. (2005), which investigated the attitudes and self-efficacy of physical education students toward including students with disabilities in mainstream classes, but which presented limitations arising from a lack of clear descriptions of the disabilities and the physical education class situations (Hutzler et al., 2005).

Given the moderating role of teachers’ perceived self-efficacy in the inclusion of students with disabilities (Ajzen, 1991; Bandura, 1997; Harter, 1985), it is necessary to develop valid and reliable instruments for measuring this concept. The SE-PETE-D scale has already been translated and adapted for various countries, such as Spain (Reina et al., 2019), Portugal (Campos et al., 2022), Mozambique (Picardo et al., 2025), the Czech (Kudláček et al., 2020), and Lithuania (Selickaitė et al., 2019), among others. From this perspective, there is a clear need to translate and validate the SE-PETE-D into Brazilian Portuguese, as the availability of this tool will enable advancements in national research aimed at identifying areas for improvement in the care of individuals with disabilities in sports projects, physical education classes, and leisure spaces.

Thus, the current study primarily aimed to translate, cross-culturally adapt, and validate the SE-PETE-D for use in Brazil. The validation process sought to confirm the suitability of the instrument for measuring the self-efficacy of physical education teachers and undergraduate students, including individuals with ID, PD, and VI, in physical education classes or sports activities. The specific objectives were to: (1) translate and cross-culturally adapt the SE-PETE-D into Brazilian Portuguese, adhering to internationally recognized guidelines for the cross-cultural adaptation of psychometric instruments; and (2) evaluate the psychometric properties of the Brazilian Portuguese version, including its content validity, construct validity, and reliability, with a sample of physical education teachers and undergraduate students.

## Methods

This project was approved by the Research Ethics Committee of the Federal University of São Paulo (CAAE: 77151624.3.0000.5505). All participants were duly informed about the study’s objectives and the procedures required to complete the questionnaires and provided written informed consent. The collected data were kept confidential and anonymous. The research design comprised two phases: the first, dedicated to translation, cross-cultural adaptation by professional translators, and content validation by experts; and the second, consisting of the application of the instrument to physical education professionals and students, aiming to collect data for Confirmatory Factor Analysis (CFA) and Exploratory Factor Analysis (EFA). These procedures were undertaken to validate the instrument for use in Brazil, providing theoretical and statistical support.

### Instrument

For the initial phase of the study, the SE-PETE-D, initially developed by Block et al. (2013), was translated into Brazilian Portuguese. Although the final published version was validated with 25 items, the present study was based on a 32-item version of the questionnaire provided by the author, Dr. Martin E. Block.

The SE-PETE-D scale has already been translated and adapted into Portuguese for use in Portugal (Campos et al., 2022) and Mozambique (Picardo et al., 2025), with both versions comprising 25 items. The present study, by contrast, adopted a methodology similar to that used for the translation into Spanish, drawing on a version provided by the original author (Reina et al., 2019). Consistent with prior translations into Portuguese, this study adopted the title “Escala de Autoeficácia na Educação Física Inclusiva—Brazil” [Self-Efficacy Scale for Inclusive Physical Education—Brazil] (EAE-EFI/Brazil), to facilitate comprehension and application of the instrument across Lusophone countries.

The scale (see attached or Supplementary material) begins with general instructions and a brief explanation of self-efficacy, based on Bandura’s (1997) theory. The instrument is then composed of four sections: the first three correspond to subscales that specifically address ID, PD, and VI. The fourth section is intended for the collection of demographic variables. Each subscale is preceded by a descriptive vignette of a student with ID, PD, or VI in a teaching situation during a physical education class, involving: (a) performing a physical fitness test; (b) teaching specific sports skills; (c) teaching the dynamics of sports games.

Each of the subscales (ID, PD, and VI) is composed of three factors related to teacher self-efficacy: (1) teaching students to help their peers with disabilities in physical education, termed “Peer Instruction” (PI), (2) modifying or adapting a task for students with disabilities, termed “Specific Adaptations” (SA), and (3) helping the student with disabilities understand what to do and to maintain focus on the task, termed “Staying on Task” (ST), or creating a safe environment during a physical education class, termed “Safety” (S) (Block et al., 2013).

The subscale referring to students with ID is composed of 11 items, distributed into the following factors: PI (3 items), SA (5 items), and ST (3 items). The subscale referring to students with PD comprises 12 items, distributed into three categories: PI (3 items), SA (6 items), and S (3 items). Finally, the subscale addressing students with VI contains nine items, allocated to the following factors: PI (3 items), SA (4 items), and S (2 items). Responses to all items are recorded on a 5-point Likert scale, ranging from 1 (no confidence) to 5 (total confidence). The final score is given by summing the responses to each item for each subscale. Higher scores indicate a greater perception of teacher self-efficacy to include students with ID, PD, or VI in physical education classes.

The final section consists of a series of demographic questions about age, biological sex (male/female), years of experience as a physical education professional (in number of years), period of the undergraduate course, for students, and information on previous training in adapted/inclusive physical education or teaching experience involving the inclusion of students with disabilities in physical education activities. In addition, there is a question about the level of experience in physical education or community sports with students with ID, PD, or VI, with the following answer options; none, little, or much.

### Procedures

The procedures for the translation, cross-cultural adaptation, and validation of the self-efficacy scale for physical education teacher education majors toward children with disabilities (SE-PETE-D) to create a Brazilian Portuguese version are described below.

The need to encompass the diverse practice settings of Physical Education professionals in Brazil (those with teaching degrees in schools and those with bachelor’s degrees working in clubs, sports initiation programs, social projects, etc.) prompted a significant adaptation to the questionnaire. To ensure the applicability of the scale to all participants, a clarifying statement was added to the initial section, explaining that the objective was to “investigate their self-efficacy in including students with ID, PD, or VI in their program, whether in physical education classes, sports training, or physical conditioning,” and that, for the purposes of this instrument, all such situations would be treated as a “physical education class”.

### Translation and cross-cultural adaptation

Given the absence of a translated and validated SE-PETE-D scale for Brazilian Portuguese, the process of translation, cross-cultural adaptation, and validation was initiated, according to the eight methodological steps proposed by Wild et al. (2005): (1) Preparation; (2) Preliminary Translation; (3) Reconciliation; (4) Back-Translation; (5) Review of the Back-Translation; (6) Cognitive Debriefing; (7) Review of the Cognitive Debriefing Results and Finalization; and (8) Review of the Final Version (Wild et al., 2005).

During the preparation stage (1), authorization and access to the 32-item version of the questionnaire were requested and obtained from the original study author, Dr. Martin E. Block. The second stage consisted of the independent translation of the original instrument into Brazilian Portuguese (the target language) by two distinct translators. In the reconciliation stage (3), the translators, initially working independently, consolidated both translated versions. Subsequently, in conjunction with the authors, the final version was refined to include the best adjustments. Back-translation (4) involved the translation of the reconciled version from Brazilian Portuguese back into the original language by an experienced third translator, unfamiliar with the original version.

The two translators and the back-translator were hired from different companies specialized in translation services. We requested that the translations into Portuguese be performed by professionals with Portuguese as their native language and that the back-translation be carried out by a professional whose native language is English. All translators were fluent in Portuguese and English, had experience in scientific translation, were independent from the research team (external to the study), and did not have access to the original document during the back-translation process.

The fifth stage (5) consisted of a review of the back-translation by Dr. Block, through a comparison between the back-translated and the original versions, in order to identify and investigate any discrepancies between the two versions. Cognitive debriefing (6) was conducted by six experienced researchers and PhDs in the area of adapted physical activity and inclusion, who evaluated the questionnaire, in order to test the comprehensibility, interpretation, and cultural relevance of the translation, as well as to determine whether the instrument was associated with the construct to be measured, in this case, the self-efficacy beliefs of physical education teachers to include students with disabilities (Kimberlin and Winterstein, 2008). Following this (stage 7), the results of the cognitive debriefing were critically analyzed to finalize the translation, through comparisons of the specialists’ interpretations, to identify and resolve any differences. Lastly (stage 8), the final translated version was reviewed to correct any typographical or grammatical errors.

### Content validity

Conceptually, an instrument can be considered valid when it possesses the capacity to measure what it purports to measure (Kimberlin and Winterstein, 2008). Content validity relates to the extent to which the items of a given instrument are associated with the construct to be measured—in this case, the perceived self-efficacy of physical education professionals in including students with disabilities. To effect this process, a mixed-methods approach was used, incorporating both qualitative and quantitative elements, for the evaluation of the questionnaire by experts in the field.

It is recommended that to conduct content validation, between four and six professionals should be involved, without exceeding a total of 10 evaluators (Lynn, 1986). Accordingly, six professionals deemed experts in the area were recruited. These evaluators were required to hold a master’s or doctoral degree in the field and possess training in inclusive physical education. Content validity was assessed online, whereby each evaluator received a form to evaluate the equivalence of each scale item to the construct of “professionals’ self-efficacy in including IWD in their classes”. Equivalence was assessed item by item, for each of the 32 items on the scale, as previously suggested (Souza et al., 2017), using a scoring system from 1 to 4, as follows:

- 1 = Item not equivalent;
- 2 = Item requires major revision to be evaluated for equivalence;
- 3 = Item equivalent, requires minor alterations;
- 4 = Item absolutely equivalent.

The form included a blank space where the specialists could insert criticisms and suggestions regarding each item. If the authors deemed it pertinent, the suggested adjustments were implemented, and the item was submitted to a new round of evaluation to verify whether the score had been altered.

### Psychometric validation

When the content validity process was completed, the translated instrument was administered to physical education professionals and students in order to assess their perceived self-efficacy in the inclusion of PWD (Venditti Júnior, 2018). This process enabled us to test the replicability of the models proposed by the authors of the questionnaire in their original version, through CFA, as well as to analyze the factor loadings via EFA. The questionnaire was administered electronically. The data were tabulated and organized in a spreadsheet using Microsoft Excel software. At this stage, to test validity, all 32 items present in the received questionnaire were considered.

The physical education professionals and students were contacted and informed about the objective of the investigation, and their participation was solicited via mobile applications, in specific parasport groups, through social media dissemination and at Paralympic sports events.

### Statistical analysis

In the current study, content validity was assessed by calculating the Content Validity Coefficient (CVC). This analysis was performed using the content validity coefficient for each item (Cvci) and the total content validity coefficient (Cvct) (Hernández-Nieto, 2002). These coefficients should exhibit values greater than 0.80 for the Cvci and above 0.80 (preferably greater than 0.90) for the Cvct (Polit and Beck, 2006). To calculate the Cvci, the following equation was used:

$\text{Cvci}=\frac{\mathrm{Mx}}{\mathrm{Vmx}}-\mathrm{pe}$

Where *M*𝑥 = ∑𝑥*i* / J, ∑𝑥*i* = the sum of the responses for each item by each judge or evaluator, *J* = the number of judges or evaluators, 𝑉𝑚𝑥 = the maximum value of the scale given to the judges, in this case 4, 𝑝𝑒 = the probability of error for each item, calculated as: 𝑝𝑒 = (1/*J*).

The Cvct was then calculated by summing each of the Cvci values and dividing by the number of items (N), as described by the following equation:

$\text{Cvct}=\frac{\sum \text{Cvci}}{N}$

EFA was conducted using a dispersion matrix based on polychoric correlations, appropriate for ordinal or non-normal data. To verify the suitability of applying EFA, the Kaiser–Meyer–Olkin (KMO) statistic and Bartlett’s test of sphericity were used, considering a value of 0.70 for the KMO index and a significance of *p* < 0.05 for Bartlett’s test of sphericity (Dziuban and Shirkey, 1974). The number of factors to be extracted was determined using the Optimized Implementation of Parallel Analysis (PA) (Timmerman and Lorenzo-Seva, 2011). Factor extraction was performed using the Robust Diagonally Weighted Least Squares (RDWLS) method, with correction for robust Chi-square and Robust Promin rotation to achieve factorial simplicity (Lorenzo-Seva and Ferrando, 2019). A Clever rotation start was used in conjunction with Weighted Varimax rotation, with 100 random starts and a maximum of 1,000 iterations, and a convergence value of 0.00001000. Factor scores were estimated based on the linear model.

In addition, a closeness to unidimensionality assessment was performed, which involves evaluating how well the scale’s three factors measure a single underlying construct: the self-efficacy of physical education professionals to include students with disabilities. For the EFA statistical analyses, the free software FACTOR was used, developed at the Universitat Rovira i Virgili in Spain by Ferrando, P. J., & Lorenzo-Seva, U., version 12.06.08 (May 13, 2025).

The quality of fit of the models proposed in the present study was evaluated in consideration of the reference framework from the original study. For model fit in CFA, the Adjusted Chi-Square (*χ*^2^/df), Root Mean Square Error of Approximation (RMSEA), Comparative Fit Index (CFI), Tucker-Lewis Index (TLI), and Bollen’s Incremental Fit Index (IFI) were considered. CFI, TLI, and IFI values above 0.90, and RMSEA values up to 0.10 were considered adequate (Bentler, 1990). The RMSEA has well-known limitations that warrant cautious interpretation. In particular, it is sensitive to sample size and model complexity and can be biased, yielding artificially high values even when other indices (CFI, TLI, IFI, SRMR) indicate acceptable fit. For this reason, we evaluated the RMSEA confidence interval alongside the point estimate and adopted an integrated interpretation of fit indices, prioritizing convergence across multiple indicators. Taking into account that *x*^2^ is very sensitive to sample size, the Adjusted Chi-Square was used, considering values below 6.0 as acceptable. For the CFA statistical analyses, JASP^®^ software was used.

## Results

After the initial phase of translation and cross-cultural adaptation had been completed, and the review of the back-translation performed and approved by the original questionnaire author, Dr. Martin E. Block, the scale underwent content validation by six experts.

### Content validity

In the assessment of content validity using the CVCI, item G from the intellectual disability subscale was the only item that presented a value (0.75) considered below the threshold of 0.8, indicative of item inadequacy. Other items received minor adjustments, such as the inclusion of a small piece of information or the substitution of a word with a synonym. The adjusted items are presented with two scores in Table 1, indicating the initial score and the final score after modification (in parentheses). The CVCT values for the subscales presented scores deemed acceptable for content validity, with values of 0.90 for the ID subscale, 0.91 for the PD subscale, and 0.90 for the VI subscale.

**Table 1:** Evaluation of content validity according to the CVR index.

Scale	Items	E1	E2	E3	E4	E5	E6	Cvci
ID Cvct 0.90	A. Stay on task—test	4	4	3	1 (3)	4	4	0.92
	B. Modify instructions—test	4	4	4	3	4	4	0.96
	C. Instruct peers to help—test	4	3	4	2	4	4	0.87
	D. Modify instructions—skill	4	4	3	4	4	4	0.96
	E. Stay on task—skill	4	3	3	3	4	4	0.87
	F. Modify equipment—skill	4	4	4	4	4	4	1.00
	G. Make modifications to skills—skill	3	4	2 (4)	2 (2)	3	4	0.87
	H. Instruct peers to help—skill	4	4	2 (3)	2 (3)	4	4	0.87
	I. Modify rules—game	4	4	3	2	4	4	0.87
	J. Stay on task—game	4	3	3	2	4	4	0.83
	K. Instruct peers to help—game	4	4	2 (2)	2 (3)	4	4	0.87
PD Cvct 0.91	A. Create individual goals—test	4	3	3	4	2	4	0.83
	B. Modify the fitness test—test	4	4	3	3	4	4	0.92
	C. Instruct peers to help –test	4	3	3	2	4	4	0.83
	D. Make the environment safe—test	3	4	4	4	4	4	0.96
	E. Make modifications to skills—skill	4	3	3	2	4	4	0.83
	F. Make the environment safe—skill	3	4	3	4	4	4	0.92
	G. Modify equipment—skill	4	4	4	2	4	4	0.92
	H. Instruct peers to help—skill	4	4	4	2	4	4	0.92
	I. Modify rules—game	4	3	4	4	4	4	0.96
	J. Modify equipment—game	4	4	4	4	4	4	1.00
	K. Make the environment safe—game	3	3	4	4	4	4	0.92
	L. Instruct peers to help—game	4	4	3	2	4	4	0.87
VI Cvct 0.90	A. Make the environment safe—test	3	4	3	4	4	4	0.92
	B. Instruct peers to help—test	4	4	3	2	4	4	0.87
	C. Modify the fitness test	4	4	3	1 (3)	4	4	0.92
	D. Modify instructions—skills	4	3	2	4	4	4	0.87
	E. Instruct peers to help—skill	4	4	3	3	4	4	0.92
	F. Make the environment safe—skills	4	3	3	4	4	4	0.92
	G. Make the environment safe—game	3	4	4	4	4	4	0.96
	H. Instruct peers to help—game	4	3	3	2	4	4	0.83
	I. Modify rules—game	4	4	3	2	4	4	0.87

Cvci, content validity coefficient for each item; Cvct, total content validity coefficient; E1, expert 1; E2, expert 2; E3, expert 3; E4, expert 4; E5, expert 5; E6, expert 6; ID, intellectual disability; PD, physical disability; VI, visual impairment.

### Psychometric validation

In total, 232 volunteers, 144 men and 81 women, completed the questionnaire for the purpose of validating the EAE-EFI/Brazil. The sample demonstrated national scope, including participants from all regions of Brazil: Southeast (178), South (12), Midwest (9), North (12), and Northeast (20), originating from 17 Brazilian states and the Federal District. The sample composition included professionals (126), with a mean of 10.6 ± 9.3 years of experience, and students (106), with a mean of 2.5 ± 1.2 years of study. Regarding experience in physical education with people with disabilities, 153 participants reported having worked or completed an internship in this area, while 79 reported no experience in this area. Regarding specific training, only 45 individuals had received some training in adapted physical activity, whereas 187 had received no specific training. Of the total volunteers, 15 identified as people with disabilities.

The descriptive statistics obtained in the EFA for each item (Mean and Confidence Interval), along with the indices of skewness, kurtosis, communality, and factor loading, are presented in Table 2 for each item of each of the three subscales. Additionally, the EFA generated results referring to the reliability of the scales. Due to the small sample size, the Goodness-of-fit Based on Latent Variables (GLB) and omega (*ω*) indicators were not considered advisable, given the possibility of presenting a positive sampling bias. Thus, the Standardized Cronbach’s alpha was selected as a measure of the reliability of the subscales, presenting values of 0.96 for the ID subscale, 0.98 for the PD subscale, and 0.97 for the VI subscale.

**Table 2:** Descriptive statistics, communalities, one extracted factor.

Item	Subscale																	
	ID (Cronbach’s *α* = 0.96)						PD (Cronbach’s *α* = 0.98)						VI (Cronbach’s *α* = 0.97)					
	*M*	Confidence interval (95%)	Skewness	Kurtosis	C	One-factor load	*M*	Confidence interval (95%)	Skewness	Kurtosis	C	One-factor load	*M*	Confidence interval (95%)	Skewness	Kurtosis	C	One-factor load
A	3.232	(3.08–3.39)	−0.048	0.005	0.765	0.823	3.526	(3.35–3.70)	−0.371	−0.453	0.854	0.876	3.368	(3.20–3.54)	−0.167	−0.500	0.740	0.827
B	3.491	(3.33–3.65)	−0.358	0.060	0.737	0.816	3.579	(3.41–3.75)	−0.243	−0.611	0.910	0.910	3.636	(3.47–3.80)	−0.522	−0.110	0.980	0.938
C	3.816	(3.66–3.98)	−0.637	0.132	0.889	0.869	3.750	(3.59–3.91)	−0.554	0.001	0.946	0.907	3.408	(3.24–3.58)	−0.261	−0.378	0.826	0.886
D	3.447	(3.30–3.60)	−0.229	−0.147	0.703	0.818	3.662	(3.51–3.81)	−0.348	0.064	0.867	0.850	3.342	(3.18–3.51)	−0.154	−0.216	0.856	0.911
E	3.211	(3.06–3.36)	−0.026	−0.290	0.902	0.895	3.461	(3.30–3.62)	−0.249	−0.346	0.763	0.866	3.570	(3.40–3.74)	−0.513	−0.106	0.937	0.942
F	3.482	(3.31–3.65)	−0.354	−0.479	0.738	0.809	3.605	(3.45–3.76)	−0.345	−0.134	0.925	0.893	3.355	(3.18–3.53)	−0.236	−0.352	0.878	0.908
G	3.425	(3.27–3.58)	−0.345	0.031	0.805	0.869	3.588	(3.42–3.75)	−0.359	−0.327	0.904	0.924	3.259	(3.09–3.43)	−0.152	−0.256	0.822	0.878
H	3.706	(3.55–3.86)	−0.468	0.085	0.953	0.909	3.768	(3.62–3.92)	−0.493	0.224	0.925	0.926	3.566	(3.39–3.74)	−0.464	−0.135	0.923	0.924
I	3.439	(3.29–3.59)	−0.325	−0.049	0.652	0.800	3.610	(3.44–3.78)	−0.507	−0.001	0.829	0.899	3.434	(3.27–3.60)	−0.278	−0.133	0.891	0.921
J	3.206	(3.06–3.35)	−0.115	0.072	0.880	0.864	3.526	(3.36–3.70)	−0.397	−0.234	0.905	0.921						
K	3.649	(3.49–3.81)	−0.504	0.167	0.880	0.854	3.561	(3.40–3.73)	−0.352	−0.145	0.884	0.890						
L							3.768	(3.61–3.92)	−0.679	0.593	0.892	0.923						

ID, intellectual disability; PD, physical disability; VI, visual impairment; C, Communality.

To verify the suitability of the data for EFA, preliminary statistical assumptions were tested. The results indicated that the data supported the conduction of the analysis. The KMO measure of sampling adequacy was greater than 0.70 for each subscale, with values of 0.85 for ID, 0.89 for PD, and 0.88 for VI (Table 3). Additionally, Bartlett’s test of sphericity was statistically significant (*p* < 0.001) across all subscales.

**Table 3:** EFA results of PE teacher separate group comparisons of the EAE-EFI Brazil and SEPET-D (Block et al., 2013).

Subscale	Item	EAE-EFI—Brazil				Block et al. (2013)			
			Factor loadings				Factor loadings		
			F1	F2	F3		F1	F2	F3
ID	A	KMO 0.85 *p* < 0.001		**0.91**		KMO 0.82 *p* < 0.001	removed		
	B				**0.89**		removed		
	C		**0.93**				**0.81**		
	D				**0.77**			**0.82**	
	E			**0.84**				**0.69**	
	F				**0.97**		removed		
	G				**0.77**		removed		
	H		**0.93**				**0.88**		
	I			***0.74***	***x***		removed		
	J			**0.97**				**0.83**	
	K		**0.98**				**0.78**		
PD	A	KMO 0.89 *p* < 0.001	0.40	−0.375	**0.83**	KMO 0.84 *p* < 0.001			**0.80**
	B		0.42	−0.356	**0.83**				**0.84**
	C		**0.98**				**0.75**		
	D			**0.80**				**0.88**	
	E				**0.72**				**0.69**
	F			**0.77**				**0.83**	
	G				**0.96**				**0.67**
	H		**0.96**				**0.92**		
	I				**0.85**				removed
	J				**0.97**				removed
	K			**0.65**	0.37			**0.80**	
	L		**0.81**				**0.93**		
VI	A	*KMO* *0.88 p < 0.001*	0.31	**0.45**		*KMO 0.73 p < 0.001*			**0.69**
	B		**0.99**	0.33	−0.369		**0.89**		
	C			***0.40***	***0.35***		**0.54**		
	D				**0.74**				**0.57**
	E		**0.84**				**0.82**		
	F				**0.94**				removed
	G			***x***	***0.98***				**0.84**
	H		**0.68**		0.37				**0.89**
	I				**0.97**		**0.84**		
	J								**0.76**

*The factor labels in the EAE-EFI—Brazil* in ID, F1 = peers’ instruction; F2 = staying on task, F3 = specific adaptations; in PD, F1 = peers’ instruction; F2 = safety, F3 = specific adaptations; in VI, F1 = peers’ instruction; F2 = safety, F3 = specific adaptations. *The factor labels in the* Block et al. (2013) in ID, F1 = peers’ instruction, F2 = staying on task; in PD, F1 = peers’ instruction; F2 = safety, F3 = specifics adaptations; in VI, F1 = peers’ instruction, F3 = specific adaptations; EFA, exploratory factor analysis; PE, physical education; KMO, Kaiser–Meyer–Olkin; ID, intellectual disability; PD, physical disability; VI, visual impairment. Bold values indicate the factor loading of an item on its correct factor; non-bold values indicate a loading on an incorrect factor. Values that are both bold and underlined indicate a loading on an incorrect factor, while an “x” marks the correct factor for the item.

Table 3 presents the independent factor structures derived in the present study and compares them with the structure presented by Block et al. (2013). The initial analysis of the items revealed high communalities, indicating that the extracted factors explain a substantial proportion of the variance in each item. Specifically, the communalities (Table 2) ranged from 0.65 to 0.95 for the ID subscale, from 0.76 to 0.95 for the PD subscale, and from 0.74 to 0.98 for the VI subscale, indicating good suitability of the items to their respective factors.

Regarding the factor loadings obtained through RDWLS analysis, most items presented satisfactory factor loadings, consistent with the proposed factors. However, some inconsistencies were identified. Item I from the ID subscale loaded onto factor F2 (Staying on Task) instead of factor F3 (Specific Adaptations), as expected. In the VI subscale, item C presented a higher factor loading on factor F2 (Safety) than it should have on factor F3 (Specific Adaptations), to which it theoretically belonged. Similarly, item G from the VI subscale did not exhibit an adequate factor loading on factor F2 (Safety), instead loading on factor F3 (Specific Adaptations) (Table 3).

In addition to these central issues, the analysis revealed that some items presented significant factor loadings on more than one factor, indicating possible factorial complexity or content overlap between the factors. These results suggest that although the scale structure is promising, certain items may require revision or adaptation to better reflect the underlying theoretical constructs in a Brazilian sample. The comparison with the original structure of Block et al. (2013) highlights the importance of considering cultural and contextual specificities when adapting assessment instruments.

### Confirmatory factor analysis for the intellectual disability subscale

The first 11 questions of the EAE-EFI/Brazil comprise the ID subscale. CFA was performed to test the factorial structure of 11 items and three factors: PI (items C-H-K), SA (items B-D-F-G-I), and ST (items A-E-J). Based on the CFA results, the proposed model exhibited an acceptable fit to the data. The adjusted chi-squared value (*χ*^2^/df) was 2.271, which falls within the range considered acceptable, given the test’s sensitivity to sample size. The CFI, TLI, and IFI indices indicated an excellent model fit, all exceeding the 0.90 threshold (Table 4).

**Table 4:** Confirmatory factor analysis indices.

Subscale	*χ* ^2^	df	*χ*^2^/df	RMSEA (90%CI)	SMR	CFI	TLI	IFI
ID	93.129	41	2.271	0.071 (0.037–0.052)	0.024	0.996	0.994	0.996
PD	321.32	51	6.300	0.144 (0.130–0.160)	0.034	0.990	0.988	0.990
PD adjusted	155,58	48	3.241	0.094 (0.078–0.111)	0.021	0.996	0.995	0.996
VI	129.19	24	5.383	0.131 (0.110–0.154)	0.025	0.995	0.993	0.995
VI adjusted	90.072	22	4.094	0.110 (0.087–0.135)	0.020	0.997	0.995	0.997

ID, intelectual disabilities; PD, physical disabilities; VI, visual impairment; *χ*^2^, chi-squared; df, degrees of freedom; *χ*^2^/df, adjusted chi-square; RMSEA, root mean square error of approximation; 90%CI, 90% confidence interval; CFI, Comparative Fit Index; TLI, Tucker-Lewis Index; IFI, Bollen’s Incremental Fit Index.

**Figure 1:**
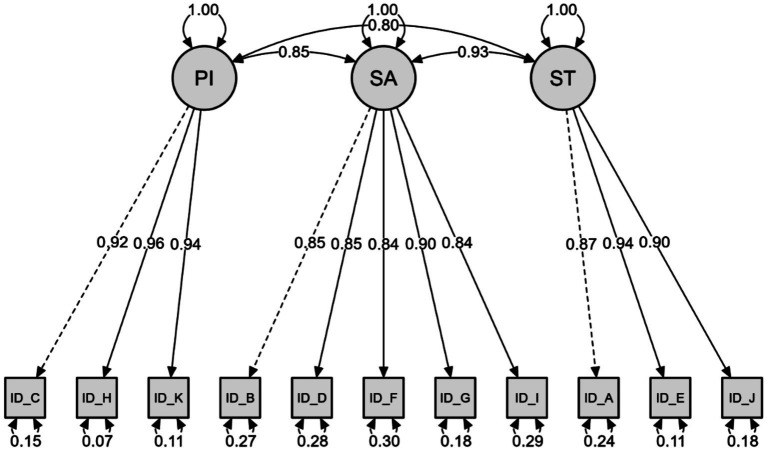
Intellectual disability subscale.

Additionally, the RMSEA (0.071; 90% CI [0.052, 0.090]) and SRMR (0.024) supported the appropriateness of the model, with values within the acceptable limits of 0.10 and 0.08, respectively. Therefore, the CFA results provide support for the validity of the proposed factor structure for the subscale in question. Furthermore, as shown in Figure 1, the model presents factor loadings of the indicators in relation to their respective factors, ranging from moderate to high (0.84–0.96), indicating a strong relationship between them. This suggests that the items are good representatives of the latent constructs. On the other hand, high correlations were observed between the factors, which may indicate that they share a considerable amount of variance. Measurement error values for each indicator were also observed, ranging from 0.07 to 0.30.

### Confirmatory factor analysis for the physical disability subscale

The following 12 questions from the EAE-EFI/Brazil comprise the PD subscale. CFA was performed to test the factorial structure of these 12 items and their three factors: PI (items C-H-L), SA (items A-B-E-G-I-J), and S (items D-F-K). The results of the CFA for the proposed model on the physical disability subscale presented an initially unsatisfactory fit to the data, as the adjusted chi-squared value (*χ*^2^/df) was 6.30 (321.321/51), exceeding the range considered acceptable. In contrast, the fit indices of CFI (0.990), TLI (0.988), and IFI (0.990) indicated an adequate fit; however, the RMSEA (0.144; 90% CI [0.130, 0.160]) and SRMR (0.034) presented values that did not align adequately with the model. To improve the model fit, adjustments were made based on modification indices, allowing covariance between the residuals of items A and B, G and J, and I and J. Following this procedure, the model exhibited improvements in fit, with the adjusted chi-squared value (*χ*^2^/df) decreasing from 6.30 to 3.241 (155.578/48), and therefore being considered acceptable. Additionally, the RMSEA reached a value of 0.094 (0.078–0.111), and the SRMR presented a value of 0.021. These values indicate a better fit of the model to the data, suggesting a more adequate representation of the scale’s factorial structure. An improvement in the CFI, TLI, and IFI indices was also observed (Table 4).

**Figure 2:**
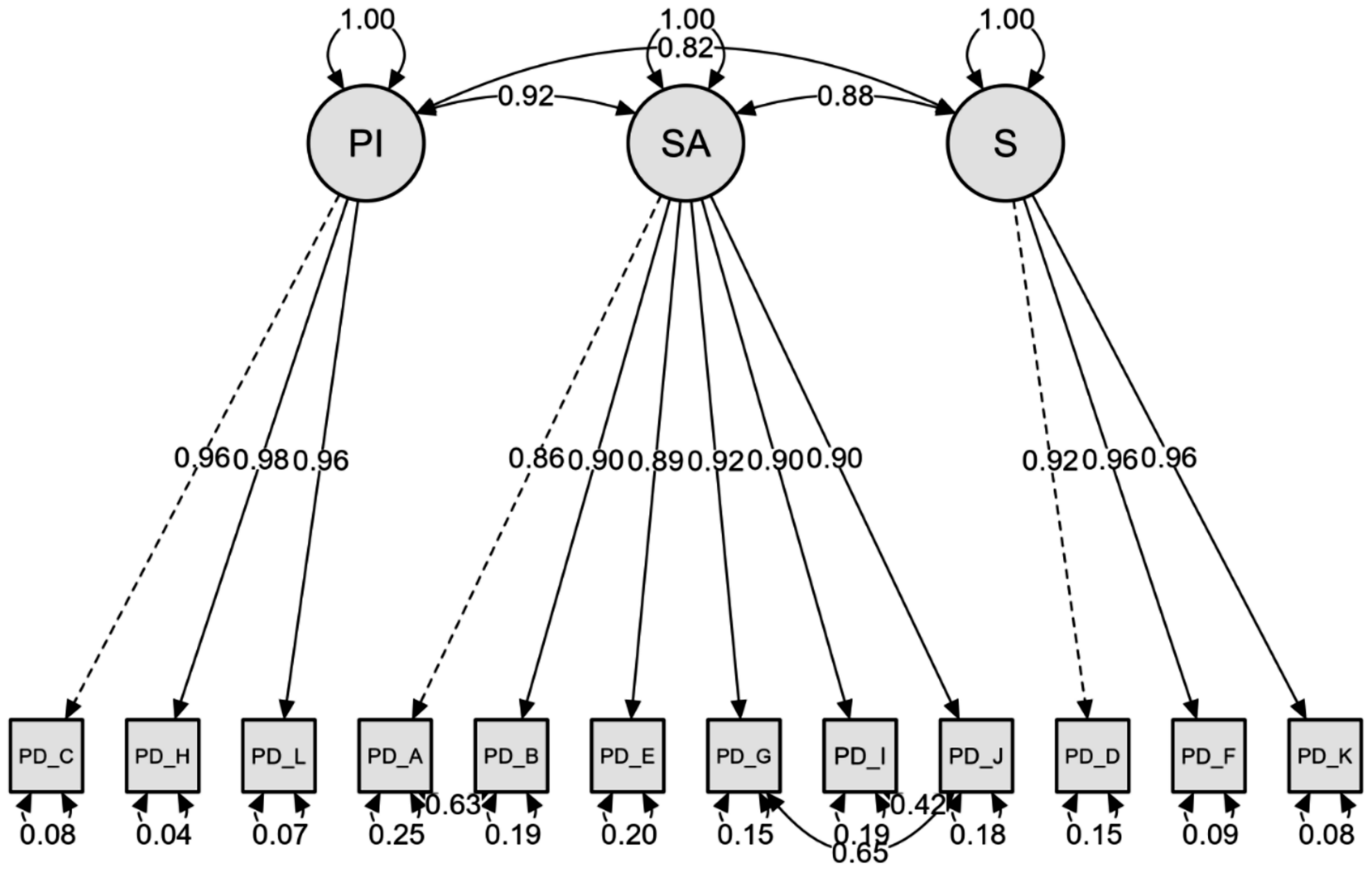
Physical disability subscale.

Figure 2 presents the factor loadings of the indicators in relation to their respective factors, varying from moderate to high (0.86 to 0.98), indicating a strong relationship with the factors. Measurement error values for each indicator were also observed, ranging from 0.04 to 0.26.

### Confirmatory factor analysis for the visual impairment subscale

**Figure 3:**
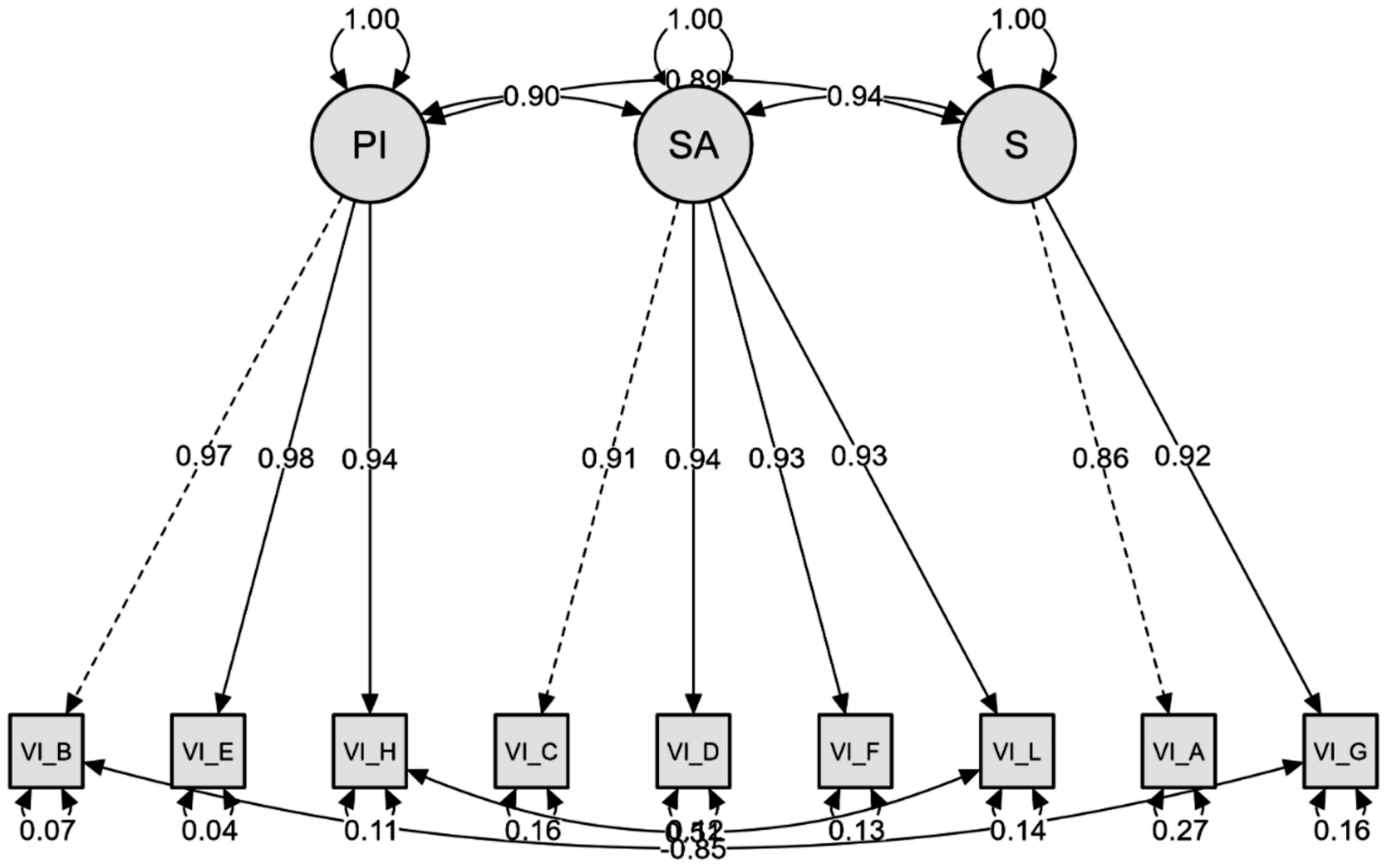
Visual impairment subscale.

The final nine questions of the EAE-EFI/Brazil comprise the VI subscale, and CFA was conducted to test the factorial structure of these nine items and three factors: PI (items B-E-H), SA (items C-D-F-I), and S (items A-G). The CFA results for the proposed model on the VI subscale presented an unsatisfactory fit, similar to PD. The adjusted chi-squared value (*χ*^2^/df) at 5.383 (129.185/24) exceeds the range considered acceptable. Although the fit indices of CFI (0.995), TLI (0.993), and IFI (0.995) indicated an adequate model fit, the RMSEA (0.131; 90%CI [0.110, 0.154]) indicated a marginal fit, and the SRMR (0.025) was at the acceptable limit. The factor loadings of the indicators varied from moderate to high (0.86 to 0.98), and the measurement errors varied from 0.04 to 0.26 (Figure 3). To improve the model fit, covariances between the residuals of items H and I, and B and G, were permitted, as indicated by the modification indices. Following these adjustments, the model showed significant improvements. The adjusted chi-squared value (*χ*^2^/df) decreased to 4.09 (90.072/22), indicating an improvement in fit. The RMSEA also decreased to 0.110 (90%CI [0.087, 0.135]), approaching the acceptable limit (Table 4). The SRMR remained low at 0.020, confirming the adequacy of the model.

This was the only subscale that presented a non-adequate RMSEA parameter, even after adjustments were made, suggesting that the model still has some limitations in perfectly fitting the observed data. On the other hand, it is worth noting that the RMSEA is sensitive to sample size and model complexity, and that in models with many parameters or with relatively smaller samples, it is common to observe values slightly above the ideal limit.

## Discussion

There is no current Brazilian Portuguese version of the SE-PETE-D scale, therefore, the objective of the current study was to undertake the translation, cross-cultural adaptation, and psychometric validation of the EAE-EFI/Brazil. The first result, obtained following evaluation by six experts, revealed that the translation of the SE-PETE-D into the Brazilian Portuguese version (EAE-EFI/Brazil) presented satisfactory CVC indices, equal to or greater than 0.90, for all three subscales evaluated. Furthermore, the EFA results indicated high reliability values, as observed by Cronbach’s alpha values (Table 2). Complementarily, the analysis of the quality of factor loadings using the EFA and CFA confirmed the validity of the Brazilian Portuguese version of the EAE-EFI; these aspects are discussed in detail hereafter.

The presented results indicate that the EAE-EFI/Brazil is a valid and reliable instrument for assessing the perceived self-efficacy of physical education professionals and students in the inclusion of students with ID, PD, and VI. Despite some limitations identified in the EFA, where item I of the ID subscale and item G of the VI subscale presented factor loadings on swapped factors, and in the CFA, where it was necessary to correlate the errors of some items of the PD and VI subscales, it is important to emphasize that the present study demonstrated robust psychometric analyses. Although future studies may present enhancements to the EAE-EFI/Brazil, as observed in other translations of the SE-PETE-D, the evidence presented herein suggests that the EAE-EFI/Brazil possesses reliable psychometric properties.

Given the relevance of the SE-PETE-D in the context of including children with disabilities in physical and sports activities, this instrument has been translated and adapted for various countries, such as Spain (Reina et al., 2019), the Czech Republic (Kudláček et al., 2020), and Lithuania (Selickaitė et al., 2019), as well as in Lusophone countries, including Portugal (Campos et al., 2022; Teixeira et al., 2025) and Mozambique (Picardo et al., 2025). In the international literature, examples such as the Spanish (Reina et al., 2016) and Portuguese (Campos et al., 2022) versions show that after initial publication of the translated instrument, psychometric data were added in subsequent versions (Reina et al., 2019; Teixeira et al., 2025). Furthermore, some other studies in different populations did not provide conclusive evidence regarding the psychometric properties or reliability of the SE-PETE-D in their translations (Jovanović et al., 2014; Tindall et al., 2016).

Among the translations cited, different approaches regarding the method and form of validation of the SE-PETE-D can be observed. For example, the versions for Portugal (Campos et al., 2022), Mozambique (Picardo et al., 2025), and the Czech Republic (Kudláček et al., 2020) chose to validate only the final 25 items of the original scale (Block et al., 2013). On the other hand, the Spanish version (Reina et al., 2019) and the Lithuanian version (Selickaitė et al., 2019) were based on a version with 33 items. The current study, in turn, used a 32-item version provided by the author, Dr. Block. Thus, it is possible to identify versions with 25 items (Portugal, Mozambique, and the Czech Republic), 29 items (Spain), 33 items (Lithuania), and our version, which has 32 items.

In the current study, as in others, we chose to maintain the original structure of the scale in the CFA, considering three factors for each of the SE-PETE-D subscales, and thereby preserving the original factorial structure for the three types of disability. The CFA parameters obtained in this work were better than those presented in the previously cited validations. Reina et al. (2019), for example, needed to reduce the number of items as a strategy to improve the fit of the scales. Our results were sufficient such that there was no need to remove any of the items, similar to the Lithuanian version (Selickaitė et al., 2019).

Considering these indicators in the present study, we identified adequate values for the goodness-of-fit indicators in the structural equation modelling analysis (CFA), including the adjusted Chi-Square (*χ*^2^/df), RMSEA, SRMR, CFI, TLI, and IFI. These results indicated a good fit of the model to the data, considering the original structure of the scale applied to the Brazilian context. The ID subscale, composed of 11 items, presented satisfactory fit indices. The PD subscale, containing 12 items, necessitated the correlation of errors for items A and B, G and J, and I and J, all belonging to the same factor (specific adaptation), which has six items in total. The VI subscale, with nine items, required correlation of the errors of items B and G, and H and I. Even while maintaining all items, the EAE-EFI/Brazil presented good regression weights for the three subscales (Figures 1–3). The values observed in the present study are higher, for example, than the values presented by the original study (ID between 0.53 and 0.87; PD between 0.58 and 0.91; VI between 0.73 and 0.93) (Block et al., 2013), as well as by the Spanish (ID between 0.61 and 0.90; PD between 0.80 and 0.95; VI between 0.73 and 0.93) (Reina et al., 2019) and Portuguese versions (ID between 0.77 and 0.93; PD between 0.80 and 0.93; DV between 0.76 and 0.96) (Teixeira et al., 2025).

In our study, as observed in other translations of the instrument (Reina et al., 2019; Selickaitė et al., 2019), correlated errors and cross-loadings were detected for some items in the CFA. A plausible theoretical explanation for the residual covariances and cross-loadings observed is the conceptual overlap among the constructs measured. Items describing ‘Specific Adaptations’ frequently include practical aspects that also affect ‘Safety’ and ‘Staying on Task’ (for example, an equipment adaptation that simultaneously enhances safety and helps the student remain focused), which can increase inter-item correlations and lead to cross-loadings. Method effects, such as similarity in item wording, item ordering, or response styles, can further inflate residual covariances. Cross-cultural adaptation may also introduce subtle semantic shifts so that items that were distinct in the source language become more similar in the target language and context. Finally, sample heterogeneity (the inclusion of both students and professionals with differing levels of experience) may accentuate these relationships. For these reasons, we permitted residual covariances only when supported by modification indices and theoretical justification. In addition, we acknowledge that the very high internal consistency observed (*α* > 0.95) may reflect item redundancy due to excessively high inter-item correlations. Future studies with larger samples should consider a careful item reduction, combining analysis of factor loadings and communalities with content review to remove redundant items, and compute alternative reliability estimates (*ω* and GLB).

In the present study, the sample included both undergraduate students and practicing professionals. This composition enhances practical representativeness, enabling assessment during training and comparisons across different teaching models and educational policies. However, it may introduce systematic variability: professionals are likely to have greater practical experience and potentially different self-efficacy perceptions than students, which can affect means, variances, and the factor structure. We recommend that future studies test measurement invariance between these groups and, where feasible, report stratified analyses to identify practical and psychometric differences between students and professionals.

The final version of the EAE-EFI/Brazil therefore comprises 32 items, distributed across three subscales: ID (11 items), PD (12 items), and VI (9 items). The scores obtained on each subscale vary according to the number of items, with lower values indicating lower perceived self-efficacy for that specific domain. Thus, the scores of the EAE-EFI/Brazil range from 11 to 55 (ID), 12 to 60 (PD), and 9 to 45 (VI). To ensure comparability of results with other studies in the international literature, especially those conducted in Portuguese-speaking countries, the authors of this study recommend that the self-efficacy indices obtained with the EAE-EFI/Brazil be reported in two forms: (a) using all 32 items of the complete scale, and (b) using only the 25 items present in the reduced version, as used in studies such as those by Campos et al. (2022) in Portugal, Picardo et al. (2025) in Mozambique, and Kudláček et al. (2020) in the Czech Republic. This approach will enable more precise comparative analyses and facilitate the integration of results in systematic reviews and meta-analyses.

## Conclusion

The current study aimed to translate, cross-culturally adapt, and validate the Brazilian Portuguese version of the EAE-EFI/Brazil, an instrument designed to assess the self-efficacy beliefs of physical education professionals and students regarding the inclusion of PWD in physical and sports activities. The results confirm that the EAE-EFI/Brazil exhibits adequate psychometric properties, demonstrating its validity and reliability for use within the Brazilian context.

Despite identified limitations, such as the need for adjustments to certain items and some divergences from prior studies, the current work represents a significant advancement, offering Brazilian researchers and practitioners an adapted and validated instrument to assess the self-efficacy of physical education professionals and students in the inclusion of PWD (ID, PD, and VI) in physical and sports activities.

The findings of this study pave the way for future research aimed at further enhancing the inclusion processes for individuals with disabilities through sport in Brazil. By using this instrument, it will be possible to explore cultural and contextual nuances that may influence the self-efficacy beliefs of professionals and students in training to include PWD in physical and sports activities. Future studies should investigate the performance of the scale across different groups of professionals and students, utilize mixed methods to deepen the understanding of educators’ perceptions, and seek evidence of validity in relation to other instruments, as well as, if possible, with performance measures.

In summary, the EAE-EFI/Brazil represents a promising tool to advance research and practice in inclusion in Physical Education, contributing to the training of more prepared and confident professionals to meet the needs of all students, regardless of their abilities or conditions.

## Data Availability

The raw data supporting the conclusions of this article will be made available by the authors, without undue reservation.
