# Supplementary material for: Translation, cross-cultural adaptation and psychometric validation of the self-efficacy scale for physical education teacher education majors toward children with disabilities for Brazilian Portuguese
Source: Front Psychol. 2026 Apr 20;17:1782205. doi: 10.3389/fpsyg.2026.1782205 (PMC13136146; doi:10.3389/fpsyg.2026.1782205)
Supplement: Supplementary file 1 [file Table_1.docx]

***Escala de Autoeficácia na Educação Física Inclusiva***

***(******EAE-EFI/Brasil)***

*Self-Efficacy Scale for Physical Education Teacher Education Majors toward Children with Disabilities (SE-PETE-D) - Brazilian Portuguese*

Renato de Carvalho Guerreiro, Ciro Winckler de Oliveira Filho, Raphael Moreira de Almeida, Martin E. Block, Andressa Silva.

Translation, Cross-Cultural Adaptation and Psychometric Validation of the Self-Efficacy Scale for Physical Education Teacher Education Majors toward Children with Disabilities (SE-PETE-D) for Brazilian Portuguese. *Frontiers in Psychology*, *xx*(x), xx-xx – 2026.

***Escala de Autoeficácia Específica Situacional e Inclusão de Alunos com Deficiência nas Aulas de Educação Física***

**Orientações:** Esta pesquisa foi elaborada para investigar sua autoeficácia em incluir alunos com deficiência intelectual, física ou visual em seu programa, seja em aulas de educação física, treinamentos esportivos ou treinamento físico, **(no presente documento, qualquer uma dessas situações serão tratadas como aula de educação física)**. Definimos autoeficácia como o seu julgamento pessoal sobre a sua competência ou a sua confiança em sua capacidade de realizar um objetivo, ou uma tarefa (Bandura, 1986). Assim sendo, queremos saber a sua opinião sobre o quanto você se sente confiante em sua capacidade de atender um aluno com deficiência intelectual, física ou visual que esteja participando de suas aulas de educação física. A escala de competência para cada pergunta vai de 1 (nenhuma confiança) a 5 (total confiança).

Não há respostas certas ou erradas, e cada profissional ou estudante de educação física responderá a essas perguntas de forma diferente. Apenas queremos saber o quanto você se sente confiante em sua capacidade de atender um aluno com deficiência intelectual, física ou visual, como os descritos abaixo, em suas aulas de educação física. A pesquisa termina com algumas perguntas demográficas. Não solicitamos seu nome ou qualquer informação de identificação, portanto, sua participação é completamente anônima.

***
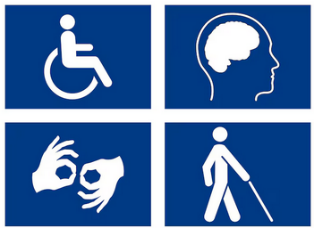
***

***Parte 1 – Deficiência Intelectual***

Abaixo, você verá a descrição de um aluno com deficiência intelectual. Na sequência, haverá uma série de perguntas sobre o quão capaz você se sente de fazer determinadas adaptações para incluir esse aluno. Em seguida, verá a descrição de um aluno com deficiência intelectual, acompanhada por outra série de perguntas. Responda a essas perguntas como se esse aluno estivesse na sua aula de educação física, na próxima semana. A escala de competência para cada pergunta vai de 1 (certeza de que não consigo) a 5 (certeza de que consigo).

********************

***Descrição de Um Aluno com Deficiência Intelectual***

João é *um aluno do ensino médio, tem 15 anos e é uma pessoa com deficiência intelectual, então, ele não aprende tão rápido como seus colegas de classe. Devido à sua deficiência intelectual, ele também não fala muito bem, então, às vezes, é difícil entender o que ele está dizendo. No entanto, ele aponta e faz gestos para ajudar as pessoas a entenderem o que ele deseja. Ele também tem dificuldade em compreender as instruções verbais, principalmente, quando possuem vários passos. João gosta de praticar os mesmos esportes que seus colegas de turma, mas não se sai muito bem quando participa de jogos reais. Embora consiga correr, ele é mais lento que seus colegas e se cansa facilmente. Ele consegue arremessar, mas não muito longe e consegue pegar as bolas lançadas diretamente para ele. Ele gosta de futebol, mas não consegue chutar a bola muito longe e nunca consegue lembrar como se posicionar na quadra. Ele também gosta de basquete, mas não possui habilidade para driblar sem perder a bola e não tem coordenação suficiente para fazer cestas. Além disso, ele não conhece bem as regras do basquete ou de outros esportes coletivos e facilmente se distrai e perde o foco durante o jogo.*

********************

Por favor, avalie o seu nível de certeza em relação a **poder realizar as ações listadas abaixo,** escrevendo o número apropriado de 1 a 5 após cada pergunta, conforme a escala abaixo.

| **1** |  | **2** |  | **3** |  | **4** |  | **5** |
| --- | --- | --- | --- | --- | --- | --- | --- | --- |
| Nenhuma Confiança |  | Baixa Confiança |  | Confiança moderada |  | Alta Confiança |  | Total Confiança |

**Perguntas A-C:** Você está realizando testes de aptidão física com os 30 alunos, entre 14 e 15 anos, da sua turma de educação física, incluindo o João.

|  |  | Confiança (1-5) |
| --- | --- | --- |
| A. | Qual o seu grau de confiança em sua capacidade de **manter o João concentrado** (“auxiliar o João na realização dos testes") durante os testes de aptidão física? |  |
| B. | Qual o seu grau de confiança em sua capacidade de **adaptar o teste para o João**? |  |
| C. | Qual o seu grau de confiança em sua capacidade de **instruir os colegas para ajudar o João** (instruir os colegas a compreenderem as limitações do João, e auxiliarem, quando necessário) durante os testes de aptidão física? |  |

**Perguntas D-H:** Você está ensinando um módulo de esporte coletivo, como vôlei, basquete ou futebol, com os 30 alunos entre 14 e 15 anos da sua turma de educação física, incluindo o João. Você está na primeira semana do planejamento e está ensinando as habilidades básicas do esporte (p. ex. a manchete, o levantamento e o saque no vôlei).

|  |  | Confiança (1-5) |
| --- | --- | --- |
| D. | Qual o seu grau de confiança em sua capacidade de **adaptar as instruções para ajudar o João** a entender o que fazer ao ensinar habilidades esportivas? |  |
| E. | Qual o seu grau de confiança em sua capacidade de **ajudar o João a se concentrar e permanecer na tarefa** ao ensinar habilidades esportivas? |  |
| F. | Qual o seu grau de confiança em sua capacidade de **adaptar os equipamentos** para ajudar o João ao ensinar habilidades esportivas? |  |
| G. | Qual o seu grau de confiança em sua capacidade de **modificar as habilidades para a realidade do João** ao ensinar habilidades esportivas? |  |
| H. | Qual o seu grau de confiança em sua capacidade de **instruir os colegas para ajudar o João** (instruir os colegas a compreenderem as limitações do João, e auxiliarem, quando necessário) ao ensinar habilidades esportivas? |  |

**Perguntas I-K:** Você está ensinando um módulo de esporte coletivo, como vôlei, basquete ou futebol, com os 30 alunos entre 14 e 15 anos da sua turma de educação física, incluindo o João. Você está na última semana do planejamento e, agora, está fazendo seus alunos jogarem jogos reais.

|  |  | Confiança (1-5) |
| --- | --- | --- |
| I. | Qual o seu grau de confiança em sua capacidade de **adaptar as regras do jogo** para que o João possa participar de forma ativa junto aos colegas? |  |
| J. | Qual o seu grau de confiança em sua capacidade de ajudar o João a **se concentrar e permanecer na tarefa** durante o jogo? |  |
| K. | Qual o seu grau de confiança em sua capacidade de **instruir os colegas para ajudar o João** (instruir os colegas a compreenderem as limitações do João, e auxiliarem, quando necessário) durante o jogo? |  |

***Escala de Autoeficácia Específica Situacional e Inclusão de Alunos com Deficiência nas Aulas de Educação Física***

***
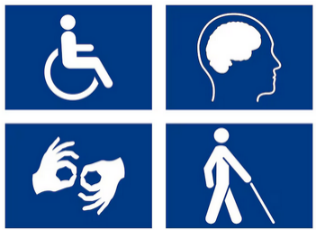
***

***Parte 2 – Deficiência Física***

Abaixo, você verá a descrição de um aluno com deficiência física. Na sequência, haverá uma série de perguntas sobre o quão capaz você se sente de fazer determinadas adaptações para esse aluno. Assim como foi feito acima, responda a essas perguntas como se esse aluno fosse estar em sua aula de educação física na próxima semana. A escala de competência para cada pergunta vai de 1 (certeza de que não consigo) a 5 (certeza de que consigo).

********************

***Descrição de Um Aluno com Deficiência Física***

*Pedro é um aluno de 15 anos do ensino médio com lesão na medula espinhal. Ele não consegue andar, então, para poder se locomover, conduz sozinho sua cadeira de rodas. Pedro gosta de praticar os mesmos esportes que seus colegas de turma, mas não se sai muito bem quando participa de jogos reais. Mesmo podendo conduzir sua cadeira de rodas, ele é mais lento que os outros alunos e fica cansado após 1-2 minutos de esforço. No vôlei, ele consegue fazer passes e saques, mas não longe o suficiente para passar por cima da rede. Ele consegue pegar bolas lançadas diretamente para ele. No entanto, ele não tem força na parte superior do corpo para arremessar uma bola de basquete com a altura suficiente para fazer cesta. Como não consegue usar as pernas, ele não consegue chutar uma bola de futebol, mas consegue empurrar a bola para frente com sua cadeira.*

********************

Por favor, avalie **o seu nível de certeza em relação a poder realizar as listadas abaixo,** escrevendo o número apropriado de 1 a 5 após cada pergunta, conforme a escala abaixo.

| **1** |  | **2** |  | **3** |  | **4** |  | **5** |
| --- | --- | --- | --- | --- | --- | --- | --- | --- |
| Nenhuma Confiança |  | Baixa Confiança |  | Confiança Moderada |  | Alta Confiança |  | Total Confiança |

**Perguntas A-D:** Você está conduzindo um teste de aptidão física com sua turma de educação física, com 30 alunos entre 14 e 15 anos, incluindo o Pedro.

|  |  | Confiança (1-5) |
| --- | --- | --- |
| A. | Qual o seu grau de confiança em sua capacidade de **criar metas individuais para o Pedro durante os testes de aptidão física?** |  |
| B. | Qual o seu grau de confiança em sua capacidade de **adaptar o teste** para o Pedro? |  |
| C. | Qual o seu grau de confiança em sua capacidade de **instruir os colegas para ajudar o Pedro** (instruir os colegas a compreenderem as limitações do Pedro, e auxiliarem, quando necessário) durante os testes de aptidão física? |  |
| D. | Qual o seu grau de confiança em sua capacidade de **tornar o ambiente seguro** para o Pedro durante os testes de aptidão física? |  |

**Perguntas E-H:** Você está ensinando um módulo de esporte coletivo, como vôlei, basquete ou futebol, com os 30 alunos entre 14 e 15 anos da sua turma de educação física, incluindo o Pedro. Você está na primeira semana do planejamento e está ensinando as habilidades básicas do esporte (p. ex. a manchete, o levantamento e o saque no vôlei).

|  |  | Confiança (1-5) |
| --- | --- | --- |
| E. | Qual o seu grau de confiança em sua capacidade de **promover adaptações nos fundamentos das modalidades esportivas** se o Pedro não conseguir ter o mesmo desempenho que seus colegas quando você estiver ensinando habilidades esportivas? |  |
| F. | Qual o seu grau de confiança em sua capacidade de **tornar o ambiente seguro** para o Pedro ao ensinar habilidades esportivas? |  |
| G. | Qual o seu grau de confiança em sua capacidade de **adaptar os equipamentos** para que o Pedro aprenda o conteúdo e as habilidades esportivas? |  |
| H. | Qual o seu grau de confiança em sua capacidade de **instruir os colegas para ajudar o Pedro** (instruir os colegas a compreenderem as limitações do Pedro, e auxiliarem, quando necessário) ao ensinar habilidades esportivas? |  |

**Perguntas I-L:** Você está ensinando um módulo de esporte coletivo, como vôlei, basquete ou futebol, com os 30 alunos entre 14 e 15 anos da sua turma de educação física, incluindo o Pedro. Você está na última semana do planejamento e, agora, está fazendo seus alunos jogarem jogos reais.

|  |  | Confiança (1-5) |
| --- | --- | --- |
| I. | Qual o seu grau de confiança em sua capacidade de **adaptar as regras do jogo** para o Pedro? |  |
| J. | Qual o seu grau de confiança em sua capacidade de **adaptar os equipamentos** para ajudar o Pedro durante o jogo? |  |
| K. | Qual o seu grau de confiança em sua capacidade de **tornar o ambiente seguro** para o Pedro durante o jogo? |  |
| L. | Qual o seu grau de confiança em sua capacidade de **instruir os colegas para ajudar o Pedro** (instruir os colegas a compreenderem as limitações do Pedro, e auxiliarem, quando necessário) ao ensinar habilidades esportivas? |  |

***Escala de Autoeficácia Específica Situacional e Inclusão de Alunos com Deficiência nas Aulas de Educação Física***

***
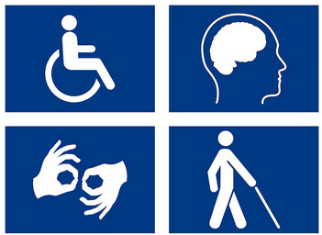
***

***Parte 3 – Deficiência Visual***

Abaixo, você verá a descrição de um aluno com deficiência visual. Na sequência, haverá uma série de perguntas sobre o quão capaz você se sente de fazer determinadas adaptações para esse aluno. Assim como foi feito acima, responda a essas perguntas como se esse aluno fosse estar em sua aula de educação física na próxima semana. A escala de competência para cada pergunta vai de 1 (certeza de que não consigo) a 5 (certeza de que consigo).

*********************

***Descrição de Um Aluno com Deficiência Visual***

*Maria é uma aluna do ensino médio. Ela tem grave deficiência visual, por isso, só consegue ver pessoas e objetos que estejam muito próximos dela. Ela gosta de atividade física e seu nível de condicionamento físico é comparável ao de seus colegas. Ela precisa de assistência física para se movimentar com segurança em ambientes de educação física. Por exemplo, ela segura o cotovelo de um colega e escuta os sinais sonoros dele, quando ela pratica corrida. Além disso, sua visão não é boa o suficiente para ver demonstrações com clareza, então, ela precisa receber instruções verbais e de alguém que a oriente durante os movimentos para entender como executar uma habilidade. Quando pratica esportes coletivos (p. ex. futebol, goalball), ela precisa que alguém fique com ela para garantir sua segurança e para saber se localizar na quadra; precisa, também, de uma bola com sinais sonoros para saber onde a bola está durante o jogo. Em relação ao seu nível de habilidade, ela não consegue pegar uma bola, mas consegue arremessar ou chutar a bola em direção a um alvo sonoro.*

********************

Por favor, avalie **o seu nível de certeza em relação a poder realizar as listadas abaixo,** escrevendo o número apropriado de 1 a 5 após cada pergunta, conforme a escala abaixo.

| **1** |  | **2** |  | **3** |  | **4** |  | **5** |
| --- | --- | --- | --- | --- | --- | --- | --- | --- |
| Nenhuma Confiança |  | Baixa Confiança |  | Confiança moderada |  | Alta Confiança |  | Total Confiança |

**Perguntas A-C:** Você está realizando testes de aptidão física com os 30 alunos entre 14 e 15 anos, da sua turma de educação física, incluindo a Maria.

|  |  | Confiança (1-5) |
| --- | --- | --- |
| A. | Qual o seu grau de confiança em sua capacidade de **tornar o ambiente seguro** para a Maria durante os testes de aptidão física? |  |
| B. | Qual o seu grau de confiança em sua capacidade de **instruir os colegas para auxiliar a** Maria durante os testes de aptidão física? |  |
| C. | Qual o seu grau de confiança em sua capacidade de **adaptar os requisitos do teste de aptidão física para a Maria** durante testes de aptidão física? |  |

**Perguntas D-H:**  Você está ensinando um módulo de esporte coletivo, como futebol ou goalball, com os 30 alunos entre 14 e 15 anos da sua turma de educação física, incluindo a Maria. Você está na primeira semana do planejamento e está ensinando as habilidades básicas do esporte (p. ex. a chute, arremesso, ou o passe).

|  |  | Confiança (1-5) |
| --- | --- | --- |
| D. | Qual o seu grau de confiança em sua capacidade de **adaptar as instruções** para ajudar a Maria ao ensinar habilidades esportivas? |  |
| E. | Qual o seu grau de confiança em sua capacidade de **instruir os colegas para ajudar a Maria** (instruir os colegas a compreenderem as limitações do Maria, e auxiliarem, quando necessário) ao ensinar habilidades esportivas |  |
| F. | Qual o seu grau de confiança em sua capacidade de **adaptar os equipamentos** para ajudar a Maria ao ensinar habilidades esportivas? |  |

**Perguntas G-I:** Você está ensinando um módulo de esporte coletivo, como futebol ou goalball, com os 30 alunos entre 14 e 15 anos da sua turma de educação física, incluindo a Maria. Você está na última semana do planejamento e, agora, está fazendo seus alunos jogarem jogos reais.

|  |  | Confiança (1-5) |
| --- | --- | --- |
| G. | Qual o seu grau de confiança em sua capacidade de **tornar o ambiente seguro** para a Maria durante o jogo? |  |
| H. | Qual o seu grau de confiança em sua capacidade de **instruir os colegas para ajudar a Maria**(instruir os colegas a compreenderem as limitações do Maria, e auxiliarem, quando necessário) durante o jogo? |  |
| I. | Qual o seu grau de confiança em sua capacidade de **adaptar as regras** para que Maria possa participar do jogo de forma ativa? |  |

***Escala de Autoeficácia Específica Situacional e Inclusão de Alunos com Deficiência nas Aulas de Educação Física***

***Parte 4 - Perguntas Demográficas***

1. __________ Idade
2. __________ Sexo Biológico
3. __________ Você é uma pessoa com deficiência?
   1. Se respondeu sim à pergunta anterior, indique qual sua deficiência: __________________________________________________________________________________
4. Em relação à Educação Física você é
5. Estudante da Licenciatura Presencial
6. Estudante da Licenciatura EAD
7. Estudante do Bacharelado Presencial
8. Estudante do Bacharelado EAD
9. Profissional formado somente na licenciatura
10. Profissional formado somente no bacharelado
11. Profissional formado em ambos
12. Qual seu nível de formação?
13. Estudante
14. Graduado
15. Especialização em andamento
16. Especialização concluída
17. Mestrado em andamento
18. Mestrado concluído
19. Doutorado em andamento
20. Doutorado concluído
21. __________ Em qual estado você atua profissionalmente ou estuda?
22. __________ Período da faculdade (para o estudante) ou ano de conclusão do curso (para o graduado).
23. __________ Você já trabalhou/fez estágio na educação física com pessoas com deficiência?
24. __________ Quantos meses você já trabalhou/fez estágio com pessoas com deficiência?
25. __________ Indique quantos cursos você já fez na área de educação física adaptada (EFA).
26. __________ Você está cursando ou possui especialização/mestrado/doutorado em EFA?
27. __________ Sua formação em EFA ofereceu/exigiu estágio/atividade prático? (sim /não)
28. __________ Se sua resposta foi sim à pergunta anterior, o estágio/atividade prática foi (**marque todas as opções que se aplicam**). Se a sua resposta foi NÃO marque a opção "Não atuei com pessoas com deficiência":
    1. ___ trabalhar individualmente com um aluno com deficiência em sua faculdade/universidade?
    2. ___ trabalhar com um pequeno grupo de alunos com deficiência em sua faculdade/universidade?
    3. ___ trabalhar individualmente com um aluno com deficiência em uma escola local?
    4. ___ trabalhar com um pequeno grupo de alunos com deficiência em uma escola local?
    5. ___ ajudar um aluno a participar de uma aula de educação física, esporte e treinamento físico?
    6. ___ realizar voluntariado em esportes comunitários como as Olimpíadas Especiais/Paralímpiadas?
    7. ___ Não atuei com pessoas com deficiência
29. Quais são suas experiências com os seguintes alunos com deficiência física, intelectual ou visual em educação física ou esportes comunitários?

Nenhuma experiência Pouca experiência Pouca experiência

Deficiência intelectual _____ _____ _____ Deficiência física _____ _____ _____ Deficiência visual _____ _____ _____

1. Quais são suas experiências pessoais com quem é portador de deficiência intelectual, física ou visual?

Membro da Alguém da Nenhuma família Um amigo escola Experiência

Deficiência intelectual _____ _____ _____ _____ Deficiência física _____ _____ _____ _____ Deficiência visual _____ _____ _____ _____

**NOTAS**

**Fatores de autoeficácia na EAE-EFI/Brasil 32 ITENS -** (dimensions of the *SE-PETE-D-Brazil*)**:**

|  | **Fatores** | | | |
| --- | --- | --- | --- | --- |
| **Subescala** | **IP** | **MT** | **AE** | **S** |
| DI | C, H, K | A, E, J | B, D, F, G, I | -- |
| DF | C, H, L | -- | A, B, E, G, I, J | D, F, K |
| DV | B, E, H | -- | C, D, F, L | A, G |

DI: deficiência intelectual (intellectual disability), DF: deficiência física (physical disability), DV: deficiência visual (visual disability), II: Instruções aos Pares (peers’ instruction), ET: Manter-se na tarefa (staying on task), AE: Adaptações específicas (specific adaptations), S: Segurança (safety).

**Fatores de autoeficácia na *SE-PETE-D* 25 ITENS -** (dimensions of the *SE-PETE-D*)**:**

|  | **Fatores** | | | |
| --- | --- | --- | --- | --- |
| **Subescala** | **IP** | **MT** | **AE** | **S** |
| DI | C, H, K | D, E, J |  | -- |
| DF | C, H, L | -- | A, B, E, G | D, F, K |
| DV | B, C, I, E | -- | A, D, G, H, J |  |

DI: deficiência intelectual (intellectual disability), DF: deficiência física (physical disability), DV: deficiência visual (visual disability), II: Instruções aos Pares (peers’ instruction), ET: Manter-se na tarefa (staying on task), AE: Adaptações específicas (specific adaptations), S: Segurança (safety).

**Referência para acessar a escala original** (Reference to acess the original scale)**:**

Block, M. E., Hutzler, Y., Barak, S., & Klavina, A. (2013). Creation and validation of the self-efficacy instrument for physical education teacher education majors toward inclusion. *Adapted Physical Activity Quarterly*, *30*(2), 184–205. <https://doi.org/10.1123/apaq.30.2.184>
